# Advancing skin cancer detection through deep learning and fusion of patient metadata and skin lesion images

**DOI:** 10.1038/s41598-025-26392-4

**Published:** 2026-01-13

**Authors:** Shafiqul Islam, Gordon C. Wishart, Joseph Walls, Per Hall, Alba G. Seco de Herrera, John Q. Gan, Haider Raza

**Affiliations:** 1 Check4Cancer Ltd., Cambridge, UK; 2 School of Computer Science and Electronic Engineering, University of Essex, Colchester, UK; 3 School of Medicine, Anglia Ruskin University, Cambridge, UK; 4 Fitzwilliam Hospital, Peterborough, UK; 5 Addenbrookes Hospital NHS Foundation Trust, Cambridge, UK; 6 School of Computer Science, National University of Distance Education (UNED), Madrid, Spain

**Keywords:** Melanoma, Predictive markers, Information technology, Computer science

## Abstract

There has been a significant rise in skin cancer incidence during the last three decades and the waiting time for skin lesion assessment in both the NHS and private sectors in the UK has increased significantly. Therefore, to reduce waiting time and to make a faster decision, there is a need to develop automated methods that can be used to classify whether a skin lesion is suspicious or non-suspicious during teledermatology triage. In this study, we propose an AI framework that uses patient metadata together with image data to classify skin lesions into suspicious or non-suspicious categories. To evaluate our proposed approach, we collected 79,246 skin lesion images along with their 22 meta-features such as lesion size, lesion colour, lesion shape, patient age, and gender from 19,295 patients who attended a network of private skin cancer diagnostic centres across the UK. We developed three separate models for skin lesion classification: (1) an AI model using only metadata that achieved 85.24 ± 2.20% sensitivity and 61.12 ± 0.90% specificity; (2) an AI model using only images that achieved 99.72 ± 1.35% sensitivity and 63.22 ± 3.11% specificity; and (3) a fused model based on both metadata and images that achieved 99.66 ± 0.28% sensitivity and 74.45 ± 0.80% specificity. The decisions of the developed AI models were then fused through a majority voting technique, which achieved a sensitivity of 99.50 ± 1.18% and a specificity of 82.72 ± 1.64%, significantly outperforming the state-of-the-art methods that rely solely on image data. Furthermore, we add a post-processing step to explain AI model decisions by implementing a soft-attention module that provides essential explainability and supports healthcare professionals in informed decision-making. The developed AI framework has great potential for the detection of suspicious skin lesions. With a reduction in patient referrals for possible biopsies, waiting times for skin cancer diagnosis and treatment will be shortened, resulting in improved outcomes.

## Introduction

The global burden of melanoma is predicted to increase to 510,000 new cases and 96,000 deaths by 2040^1^. However, NMSC accounts for 90% of all malignant skin tumours, including BCC and SCC. The incidence of NMSC increased by 33% between 2007 and 2017 to reach 7.7 million cases worldwide^2^. Cancer Research UK reports 16,744 new cases of melanoma per year in the UK^3^ and 155,985 cases of non-melanoma^4^, although it is widely acknowledged that the number of non-melanoma cases is an underestimate. Against this growing incidence of skin cancer globally, there is a national and international shortage of dermatologists^5,6^, specialist skin cancer nurses, and telemedicine reporters. As a result, the waiting time for skin lesion assessment and diagnosis has significantly lengthened since the end of successive lockdowns during the COVID-19 pandemic. The development of advanced models to assist with lesion classification during teledermatology triage could significantly reduce the waiting time for skin cancer diagnosis and treatment.

In the early 2000s, skin cancer was diagnosed using conventional techniques such as the 7PCL that often showed moderate performances (sensitivity 73.3%, specificity 57.1%)^7^. Studies such as^8,9^ explored the potential of introducing computer-based methods for skin cancer diagnosis. These researchers concentrated on analysing dermoscopic skin lesion images, initially segmenting the lesion and then extracting handcrafted features, which were used to develop basic ML models. Image segmentation was performed using a series of thresholding algorithms^10^. Features such as shape, texture, and colour were then calculated from the segmented lesion. These features were used to train ML classifiers, KNN, and SVM models. With technological advancements and the availability of open-source datasets, DL models, such as CNN, have since been used to analyse skin lesion images.

Brinker et al.^11^ reviewed 13 publications that had implemented deep CNNs pre-trained on millions of images. One of the key papers from this review^12^ compared the performance of CNNs versus 21 board-certified dermatologists. They employed an InceptionV3 network pre-trained on an extensive dataset of over 120,000 images, encompassing approximately 757 different skin diseases. The model was eventually used to differentiate between benign and malignant lesions, achieving performance comparable to that of dermatologists. A similar work^13^, also compared the performance of a CNN with dermatologists of all levels of experience (Junior to Chief Physicians). They used the ISIC 2016 dataset^14^ and implemented a pre-trained ResNet50 model to classify typical nevi from melanomas, which outperformed 136 out of 157 dermatologists. Another study^15^ employed VGGNet on the ISIC 2016 dataset. They achieved 81% accuracy with the fine-tuning method. In line with the above,^16^ emphasised the intra-class variations and inter-class similarities between different types of lesions, which make it extremely difficult for machines to differentiate between them. At first, they attempted to narrow down the area of analysis by randomly cropping the images from the centre in different ratios. They also implemented several image augmentation techniques to increase the size of the dataset. They proposed a variation of the ResNet model by replacing the residual blocks with attention residual blocks. Compared to the top 6 neural network submissions to the 2017 ISIC challenge^17^, their proposed model achieved the highest overall AUC of 91.70%^18^. In recent years, researchers have also utilised ViTs for skin cancer detection, achieving state-of-the-art performance^19,20^. However, training ViTs on limited datasets remains an ongoing challenge.

A recent study^21^ took a different approach. Instead of utilising dermoscopic images, they used skin lesion images captured via a smartphone through an in-house developed Mobile App. In this research, they highlighted the importance of the patient’s clinical information by comparing the performance of models based solely on images versus those combining images with patients’ clinical details. They collected eight clinical features, including patient age, location of the lesion on the body, whether the lesion itches, bleeds or has bled, causes pain, has recently grown, has changed in pattern, and if it has an elevation. Several CNNs, including GoogleNet, ResNet, VGGNet, and MobileNet, were used to extract features from lesion images. For a comparative analysis, these extracted features were combined with the clinical information and fed into an ML classifier for inference. They observed a 7% overall increase in balanced accuracy when clinical information was included in the analysis. Similarly, the study in^22^ evaluates various combinations of dermoscopic, macroscopic, and clinical metadata for binary melanoma and multi-class cancer detection, finding that combining all three yields the highest overall AUC of 88.80%. Additionally, the study in^23^ assessed the use of clinical information alone by analysing data from the National Health Interview Survey (NHIS) from 1997 to 2015 to classify non-melanoma skin cancers against “never-cancer” skin conditions. This dataset includes patient details such as the patient’s age, body mass index (BMI), ethnicity, and several lesion characteristics from over 450,000 patients’ skin lesions. They employed a basic feed-forward neural network for binary cancer status classification, achieving an AUC of 81% with 86.2% sensitivity and 62.7% specificity on the validation set.

In our previous study^24^, we investigated the potential of patient metadata in skin cancer detection. We identified a new list of seven risk factors named *“Check4Cancer (C4C) risk factors”* from a pool of 22 meta-features responsible for the development of all skin cancer subtypes (melanoma, SCC, BCC) through an ensemble of five AI models, which significantly outperforms the existing 7PCL and Williams methods with a balanced accuracy of 71.27% and sensitivity of 80.46%. We also proposed a new skin cancer risk score named *“C4C risk score”*, which is based on the weighting of *“C4C risk factors”* with weights determined by intelligent data analysis. Using the *“C4C risk score”* alone can achieve 68.90% balanced accuracy and 76.09% sensitivity in classifying suspicious and non-suspicious skin lesions, significantly higher than the existing 7PCL^7^ risk score and Williams risk score^25^. Furthermore, we fused the *“C4C risk factors”* with the 7PCL and Williams risk factors to find the best feature combination, which achieves the highest overall performance with a balanced accuracy of $73.18\%$ and a sensitivity of $85.24\%$. In this study, we investigate the fusion of the newly identified skin risk factors and weighted risk score together with lesion images using DL models to further boost the performance of skin cancer detection.

Dermoscopic images are now widely used for skin cancer detection^26^. AI-based early skin cancer detection is an active area of research and has achieved the state-of-the-art performance^27^. In the literature, the majority of previous skin cancer classification research has focused solely on image data alongside DL models, with limited exploration of detecting skin cancer through the fusion of patient metadata and image data. While studies such as^21–23^ incorporated a limited set of patient metadata (age, gender, and anatomical location), they did not highlight the significance of combining patient metadata with image data for enhancing AI model performance. In an attempt to fill the mentioned research gap and to further improve the skin cancer detection performance through utilising patient metadata and image data altogether, we devised an AI framework for classifying fused metadata and skin lesion images into suspicious or non-suspicious categories. This research work has made the following major contributions:

1. Collection and evaluation of 79,246 skin lesion images and metadata from 19,295 patients across a national network of private UK skin diagnostic clinics. For each lesion, we collected 22 meta-features and two types of images using DER images and DSLR images. We identified a new set of seven primary risk factors, including lesion pinkness, lesion size, lesion colour, lesion inflammation, lesion shape, lesion age, and natural hair colour. These factors are pertinent not only for melanoma but for all types of skin cancer.
2. Development of a multi-modal AI framework, which combines patient metadata with skin lesion images for the classification of concerning skin lesions. Three types of AI models have been developed by varying input data types. Ultimately, the model utilising both metadata and images achieved the highest performance with 99.66 ± 0.28% sensitivity and 74.45 ± 0.80% specificity, significantly higher than the AI model performance using image data only (99.72 ± 1.35% sensitivity and 63.22 ± 3.11% specificity). Furthermore, we fused outcome decisions of the developed AI models through a majority voting technique, which achieved a sensitivity of 99.50 ± 1.18% and a specificity of 82.72 ± 1.64%, significantly outperforming the state-of-the-art methods that rely solely on image data.
3. A post-processing module consisting of Grad-CAM and soft attention technique is added into our AI framework to enhance decision-making transparency by generating heat maps of skin lesions, providing crucial AI explainability to support healthcare professionals in their decision-making process.

## Methods

### Data collection

We have collected 79,246 images from 39,623 skin lesions belonging to 19,295 patients who attended C4C’s UK network of private skin cancer diagnosis clinics between 2015 and 2022, as summarised in Table 1. For each skin lesion, we collected two types of images, one using a dermoscopic camera (39,623 images) and another using a DSLR camera (39,623 images).

**Table 1:** Skin lesion image data collection summary.

No. of patients	No. of lesions	No. of images	Suspicious	Non-suspicious	Time period
19,295	39,623	79,246	11,258	67,988	2015–2022

**Fig. 1:**
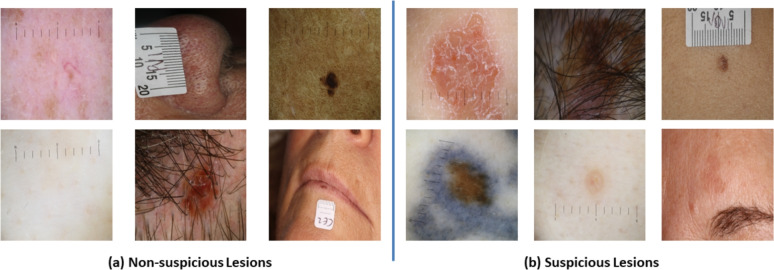
Collected skin lesion images: (**a**) non-suspicious lesions and (**b**) suspicious lesions.

**Fig. 2:**
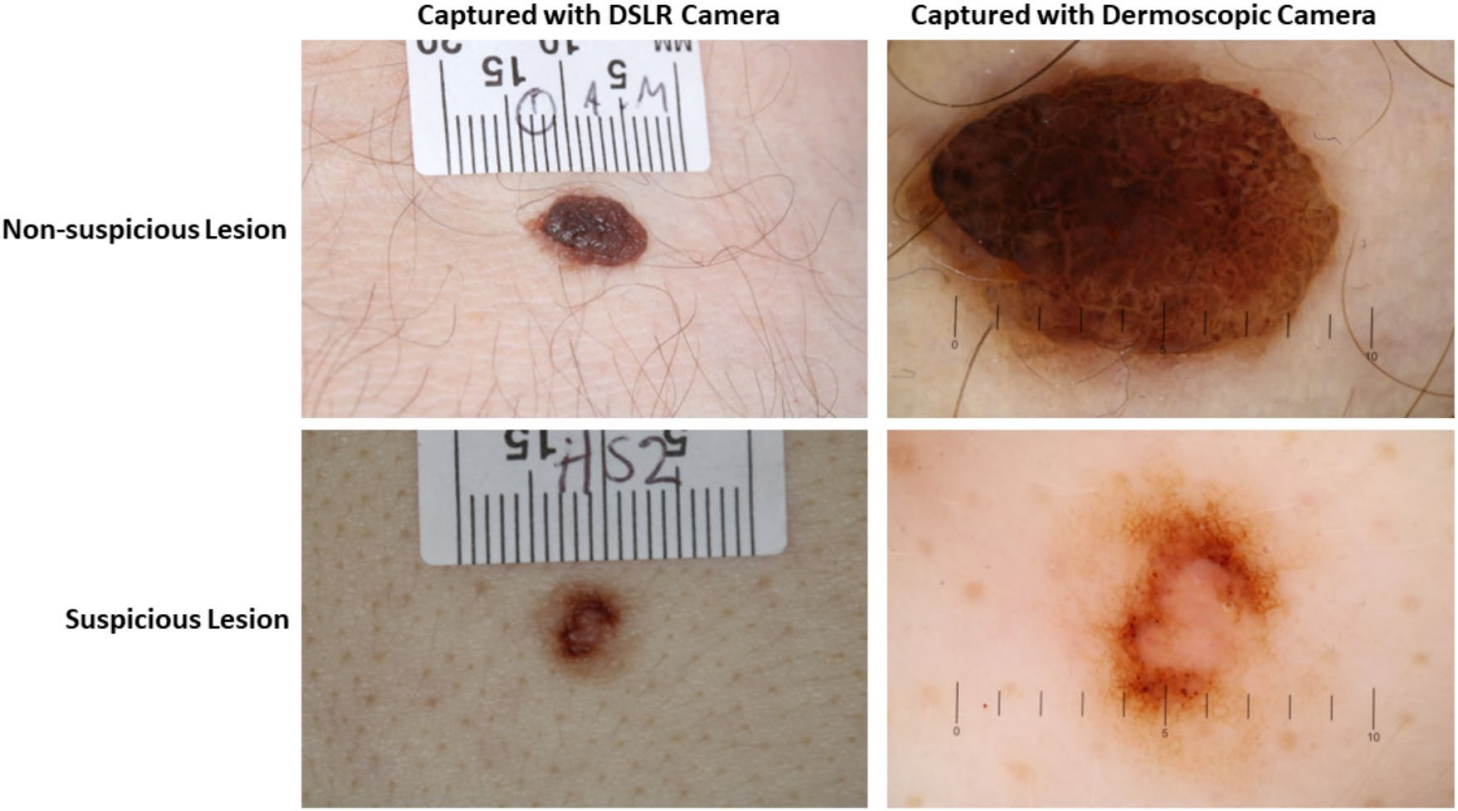
Two types of cameras (dermoscopic and DSLR camera) are used to capture images of suspicious and non-suspicious skin lesions. A total of 79,246 skin lesion images along with their corresponding patient metadata, have been analysed in this study for skin lesion classification into suspicious and non-suspicious categories.

A snapshot of the skin lesion images belonging to suspicious and non-suspicious categories are shown in Fig. 1, whereas pairs of skin lesion images captured by dermoscopic and DSLR cameras are illustrated in Fig. 2. For each lesion, we also collected 22 meta-features, with further details described in our previous study^24^. The skin type values were not availablein our dataset during the development of the AI model. Our dataset included UK patients, the majority of whom comprise Fitzpatrick skin types I—IV as mentioned in a UK-based similar study in^28^, and the results may not be applicable to Fitzpatrick skin types V and VI due to their under-representation in the dataset. The patients were informed that collected data might be used for research, and the data was anonymised to ensure confidentiality. Informed consent was obtained from the patients. All experimental protocols were approved by the University of Essex Research Ethics Committee on 8th February 2023 (Ref. No: ETH2223-0619). Following approval, anonymised lesion images and clinical metadata were transferred to C4C’s secure server for AI model development. C4C is a private healthcare company registered in the UK that provides cancer screening and diagnostic services for skin cancer patients. C4C has permission to use the collected data and holds ISO 27001 and Cyber Essentials certification, as they are accustomed to handling personal and medical (special category) data. C4C is fully compliant with UK data protection legislation and the duty of confidentiality. All methods were carried out in accordance with relevant guidelines and regulations.

Each lesion was visually assessed by the in-house skin cancer specialists during teledermatology triage. The telemedicine reporters were both skin cancer surgeons who have both extensive experience in telemedicine reporting (>15 years) and extensive research experience in skin cancer diagnosis. The experts classified pigmented lesions with atypical features in size, shape, colour, or dermoscopic appearance of melanoma as suspicious. Furthermore, skin lesions suspicious of either BCC, SCC, potentially pre-malignant Actinic Keratoses, Bowen’s disease, or in-situ carcinoma were also rated as suspicious. We considered the experts’ classification (suspicious or non-suspicious) as ground truth while developing AI models. However, to have a fair comparison with the existing models, we only considered biopsy proven cancer cases to evaluate the developed AI model. There are 1546 Melanoma cases, 4420 BCC cases, 530 SCC cases, and 4762 other suspicious cases belonging to suspicious categories. Conversely, a significantly higher number of images (43,987) with a subcategory reported as Mole belongs to the non-suspicious group. Other major subcategories comprise naevus (1829 cases), actinic (1918 cases) and Seborroehic keratosis (8778 cases).

There are 67,988 non-suspicious images (out of 79,246) and 11,258 suspicious images, which were collected from diverse locations of lesions to help build a comprehensive AI model. Although the dermoscopic camera provides magnified and better quality images than the DSLR camera, both camera images were used to build a robust AI model that is capable of classifying both types of images when implemented in real-world applications. Five images were discarded due to inadequate lighting (2), image corruption (2) or low resolution (1).

**Fig. 3:**
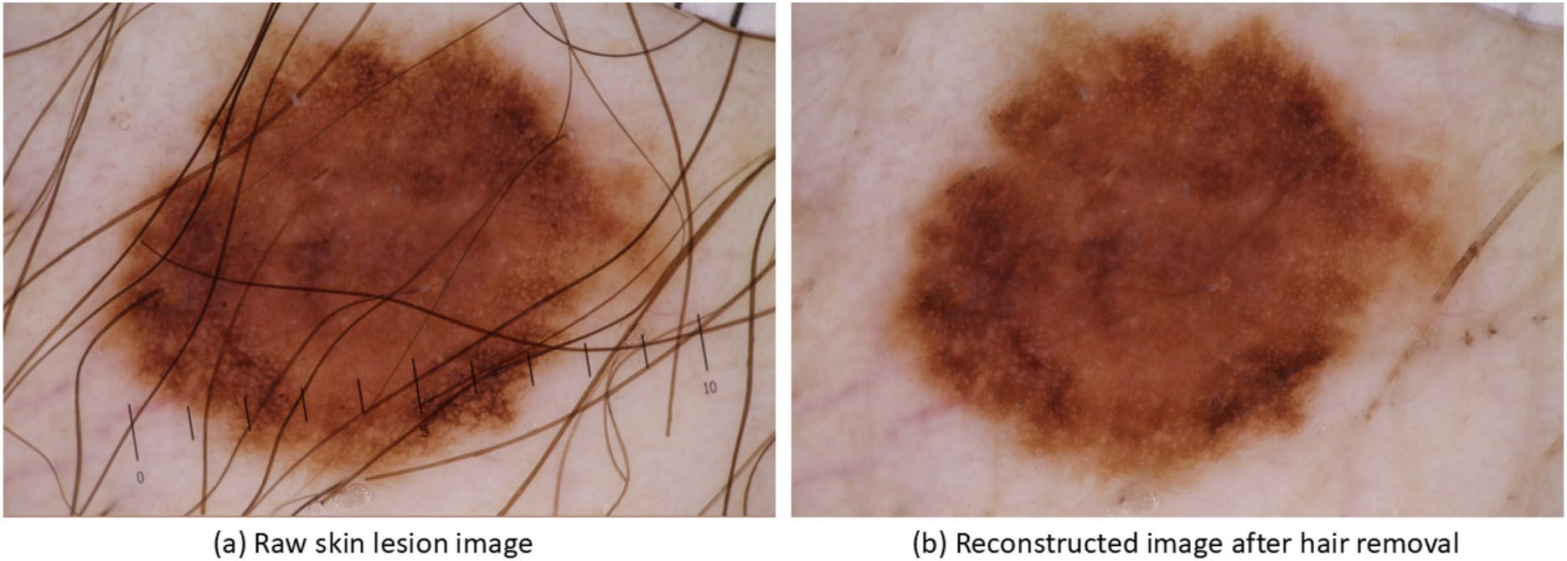
Reconstruction of skin lesion images after hair removal.

### Data pre-processing

There are artefacts, such as hairs and rulers, present in the skin lesion images (as shown in Fig. 1), which we believe could potentially reduce the AI model’s performance. To address this, we implemented the hair removal method used by Bardou et al.^29^. While this method effectively removes hairs from the skin lesion images, it does come with a trade-off, as the quality of the reconstructed images appears to diminish upon visual inspection.

An example of the pre-processed skin lesion images after hair removal is presented in Fig. 3. By visual inspection, we observed that hairs were removed and the reconstructed images had better lesion area clarity, which helped to correctly classify lesions into suspicious or non-suspicious categories.

**Fig. 4:**
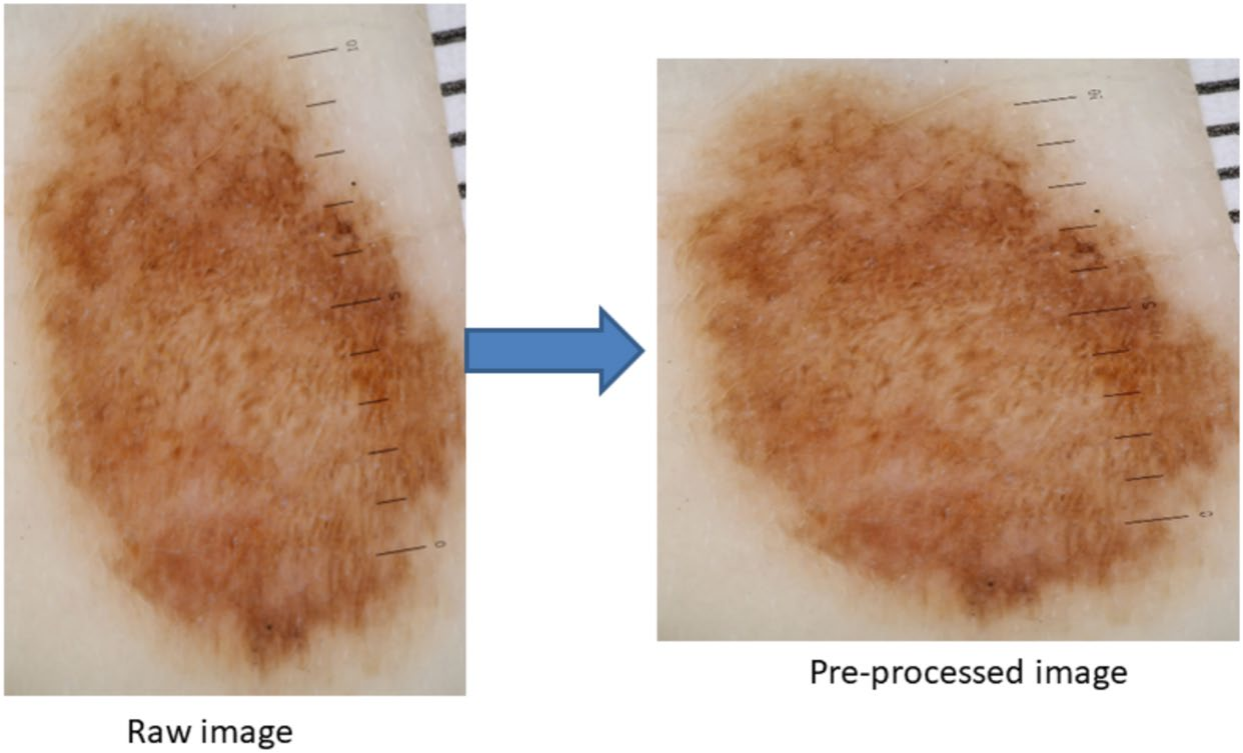
Reshaping of skin lesion images. Raw image size: 3.39MB, resolution: 2848 × 4273 pixels, pre-processed image size: 187KB, resolution: 1024 × 1024 pixels. Through a visual inspection, we anticipated that the lesion shape had been distorted after resizing.

**Fig. 5:**
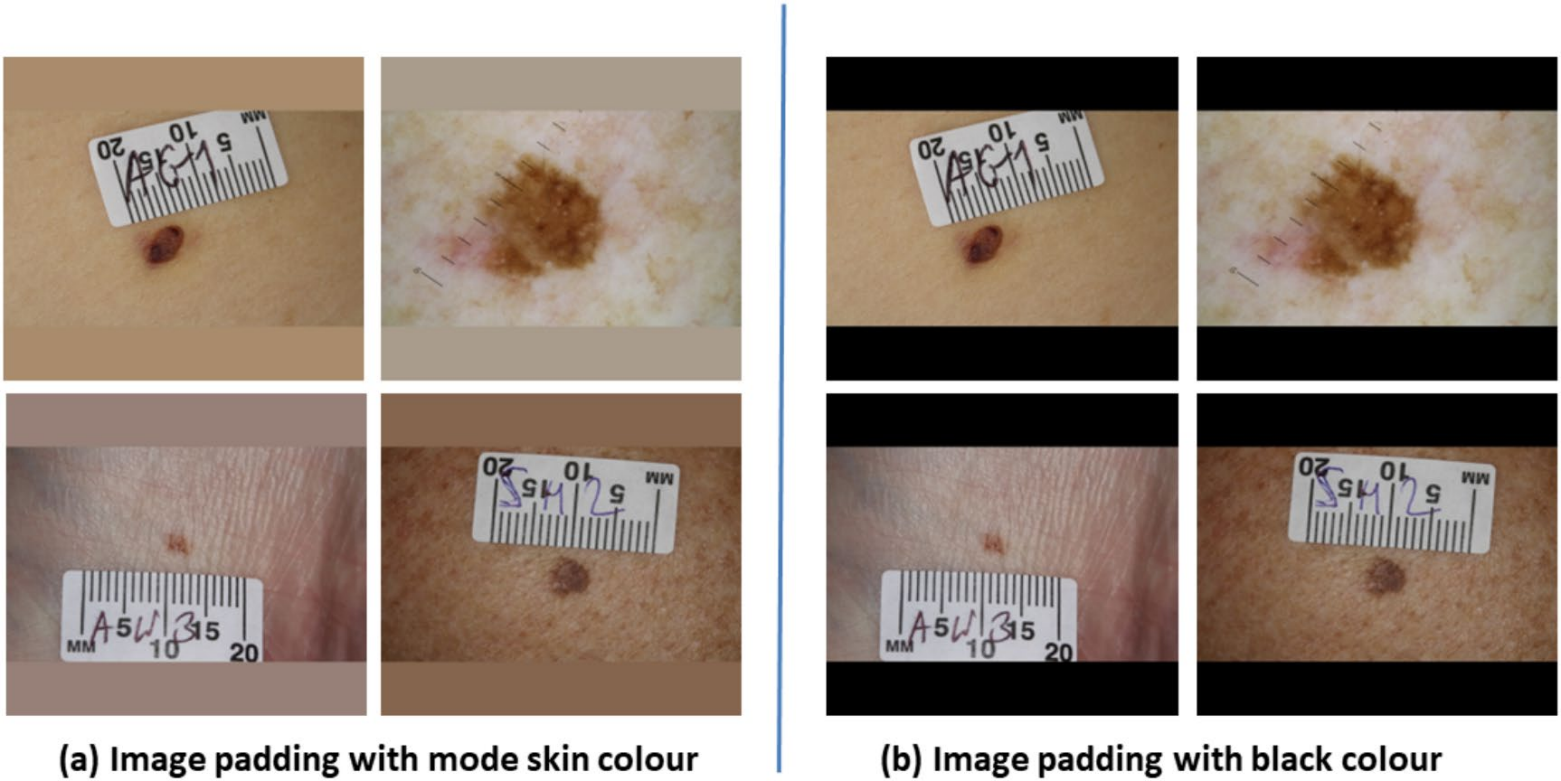
Padding approaches tested for image reshaping.

The average size of the collected raw skin lesion images is approximately 5MB with a resolution of 3000 × 4000 pixels. To build the AI model, we resized the images and converted them to a square shape with a resolution of 1024 × 1024 pixels. Reshaping images in this way allows for training an AI model with lower memory requirements and reduced training time compared to using raw images alone. Additionally, it is recommended to provide square-shaped images as input to the AI model, as this enables more efficient convolutional computations compared to non-square images^30^.

However, this process distorts the original shape of the lesion, which is a crucial feature for accurately classifying lesions into suspicious or non-suspicious categories as highlighted in Fig. 4. Therefore, we adapted and tested two different image reshaping approaches as shown in Fig. 5a reshaping with skin tone colours where pixel values of padding location were replaced with mode pixel values of the corresponding images; b) reshaping with black padding, where pixel values of padding location were replaced with (0,0,0) values. These padding methods were compared to identify the right approach that yielded the best AI model performance, with the aim of achieving optimal performance in skin cancer detection.

### AI model development

**Fig. 6:**
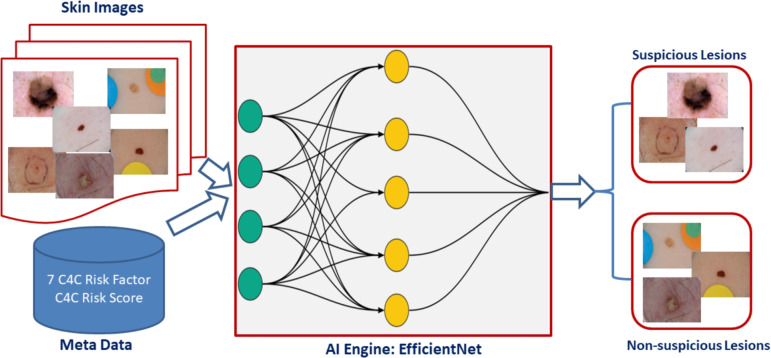
The proposed AI framework for skin lesion classification into suspicious vs non-suspicious groups based on metadata (C4C risk factors and C4C risk score) and images.

An overview of the proposed multi-modal AI framework for suspicious skin lesion classification is outlined in Fig. 6. During model development, raw images were pre-processed to re-shape to 1024 × 1024 pixels and metadata were encoded to convert from string to nominal and then fed as input to the AI model. The C4C risk factors, together with the C4C risk score and skin lesion images, are all used as inputs to the AI model, and the model provides a decision whether the input belongs to the suspicious or non-suspicious group.

**Fig. 7:**
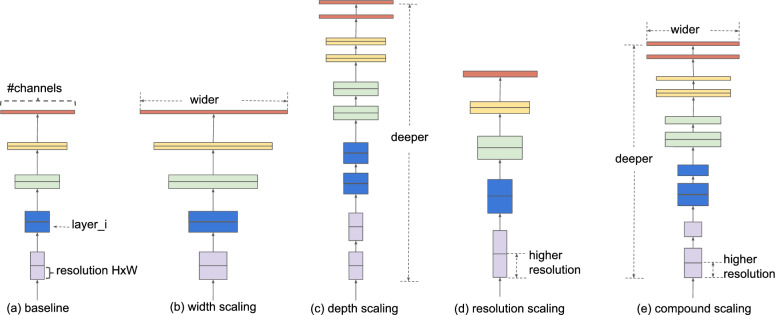
Compound scaling of EfficientNet can accommodate different image sizes to gain better performance: (**a**) is a baseline network example; (**b**–**d**) are conventional scaling that only increases one dimension of network width, depth, or resolution. (**e**) Our method is adapted from the study^31^, a compound scaling method that uniformly scales all three dimensions with a fixed ratio.

**Fig. 8:**
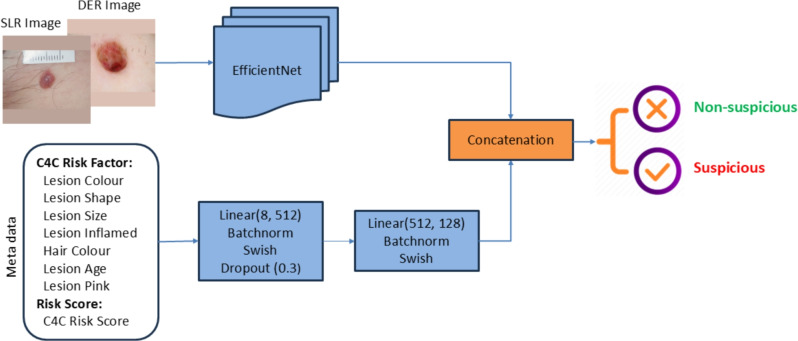
Fusion of metadata with image data to build an AI framework.

#### AI engine

EfficientNet–B2 was used as the backbone AI model architecture, having been developed by Google^31^ as a more efficient method that delivers state-of-the-art model performance that outperforms other models by using a compound scaling method. EfficientNet can accommodate different image sizes to gain better performance as shown in Fig. 7a is a baseline network example; (b–d) are conventional scaling that only increases one dimension of network width, depth, or resolution. (e) Our method is adapted from the study^31^, a compound scaling approach that uniformly scales all three dimensions with a fixed ratio. In developing the AI model, we used ensembling (merging multiple model outputs to improve model performance) of the EfficientNet AI model for classifying input data into a binary class (suspicious vs non-suspicious). The metadata and images were divided into training (80%) and test (20%) sets. The training was used to build the AI model and to find optimal weights. Then the developed model was evaluated on test data. In small to medium-sized datasets, image augmentation is important to prevent overfitting. In our pipeline, we used the following augmentations from the popular and powerful Pytorch augmentation library Albumentations^32^: Transpose, Flip, Rotate, RandomBrightness, RandomContrast, MotionBlur, MedianBlur, GaussianBlur, GaussNoise, OpticalDistortion, GridDistortion, ElasticTransform, CLAHE, HueSaturationValue, ShiftScaleRotate, and Cutout. Pytorch Albumentations is available off the shelf and efficiently implements a rich variety of image transform operations that are optimised for performance for different computer vision tasks, including object classification and detection.

For the training schedule, we employed cosine annealing with a warm-up phase lasting one epoch. The AI models were trained for a total of 50 epochs. The initial learning rate for the cosine cycle was adjusted for each model, ranging from 1e-4 to 3e-4. During the warm-up epoch, the learning rate was set to one-tenth of the initial rate for the cosine cycle. A batch size of 32 was used across all models.

The computational machine we used for AI model development has: RAM (128GB); CPU (Intel ®$\textrm{Core}^\textrm{TM}$ i9 18 Core Processor, 3.0GHz); GPU (24GB NVIDIA GEFORCE RTX 4090). We used NVIDIA CUDA CUDNN, Python 3.7, PyTorch, and Anaconda packages and tools to develop AI models.

#### Multi-modal data fusion

The key advantage of our approach to developing an AI model for skin lesion classification lies in the availability of metadata for the 79,246 images. This metadata includes eight meta-features, comprising seven C4C risk factors and the overall C4C risk score, which can be integrated with image outputs to enhance model performance. We explained in detail about the identification of seven C4C risk factors and the C4C risk score in our previous study^24^. The integration of metadata with images is illustrated in Fig. 8 adapted from the study^33^. In this context, ‘Swish’ is an activation function^34^, and ‘concat’ refers to the concatenation or fusion of the image vector with the metadata vector, followed by a linear dropout layer with a ratio of 0.5 to get the final feature map to classify whether the input belongs to suspicious or non-suspicious categories.

#### AI model decision fusion

**Fig. 9:**
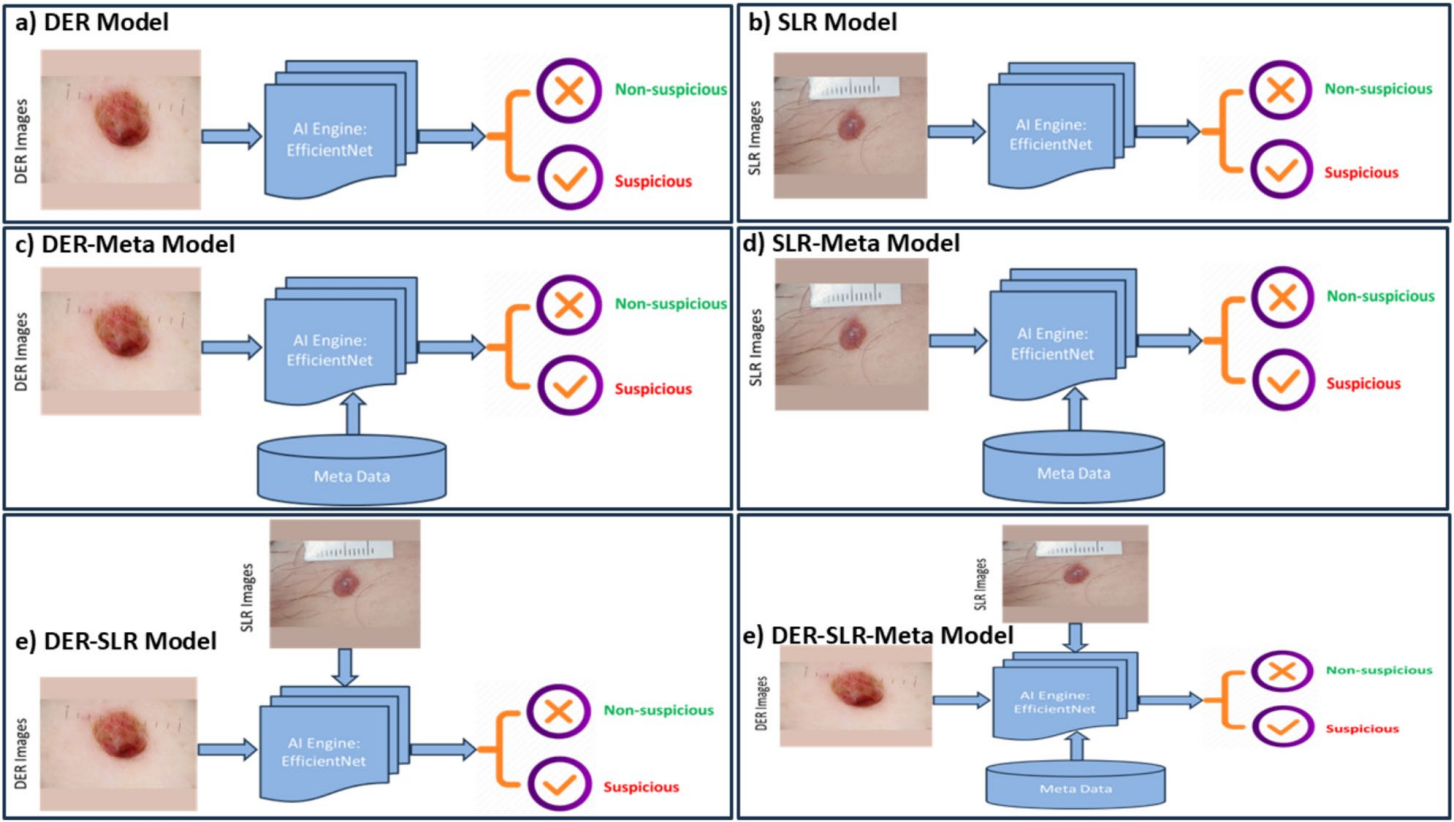
Development of a multi-modal AI framework which combines patient metadata with skin lesion images to classify suspicious skin lesions. A total of six AI models have been adapted and fused by varying input data types: (**a**) DER, (**b**) SLR, (**c**) DER+metadata, (**d**) SLR+metadata, (**e**) SLR+DER, and (**f**) SLR+DER+metadata.

For decision fusion, we have adapted six AI-based EfficientNet–B2 models using different combinations of input data, as shown in Fig. 9. We used the same configuration of the base EfficientNet–B2 model, nevertheless only varied the input data. The fused six models are briefly summarised as-

- EfficientNet–B2–DER: the collected DER images were used as input to develop this AI model.
- EfficientNet–B2–SLR: the collected SLR images were used as input to develop this AI model.
- EfficientNet–B2–DER–Meta: We fused DER images along with the metadata to be used as inputs during this particular model development.
- EfficientNet–B2–SLR–Meta: We fused SLR images along with the metadata to be used as inputs during this particular model development.
- EfficientNet–B2–DER–SLR: We used both DER and SLR images as inputs during the model development.
- EfficientNet–B2–DER–SLR–Meta: we fused DER and SLR images along with the metadata to be used as inputs during this particular model development.

The outcomes from these AI models on test data were fused based on majority voting to get a final decision whether the test input belongs to the suspicious or not suspicious category.

#### Data split and evaluation metrics

The 79,246 skin lesion images were divided into training and test datasets, with 80% allocated to training and 20% to testing. The dataset was split for training and testing randomly; patients’ characteristics (age, gender) were not considered. To avoid data leakage, all data (including images and corresponding metadata) for each patient was exclusively assigned to either the training or testing dataset. The training data was used to build the models. These models were optimised by fine-tuning hyperparameters, and the best-performing models were selected based on their results using the training data. The chosen models were then evaluated using the test dataset. The performance of the AI framework was measured using the sensitivity, specificity, ACC, and AUC evaluation metrics:

1 $$\begin{aligned} \textrm{Sensitivity} = \frac{TP}{TP + FN} \end{aligned}$$

2 $$\begin{aligned} \textrm{Specificity} = \frac{TN}{FP + TN} \end{aligned}$$

3 $$\begin{aligned} \textrm{ACC} = \frac{SEN + SPC}{2} \end{aligned}$$

4 $$\begin{aligned} \mathrm {\text {AUC}} = p(Score(TP) > Score(TN)) \end{aligned}$$

where *TP*, *TN*, *FP*, *FN* refer to true positive (suspicious classified as suspicious), true negative (non-suspicious classified as non-suspicious), false positive (non-suspicious misclassified as suspicious), and false negative (suspicious misclassified as non-suspicious) instances, respectively. The AUC of a classifier is the probability that a randomly chosen TP case will be ranked higher than a randomly chosen TN case.

**Fig. 10:**
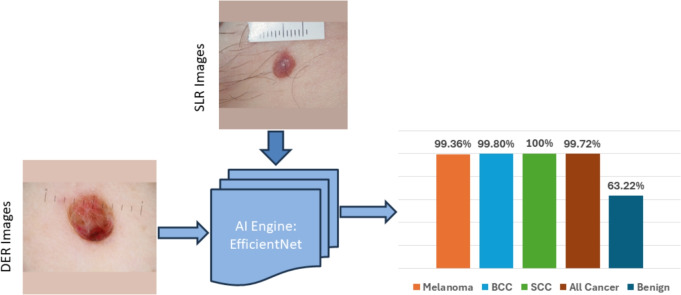
Performance of the AI-based model using image data alone.

## Results and discussion

**Table 2:** Performance of the AI-based model using metadata alone.

Method	Risk factor	Sensitivity	Specificity	ACC	AUC
7 C4C risk factors	1. Lesion Colour, 2. Lesion Shape, 3. Lesion Size, 4. Lesion Inflamed, 5. Hair Colour, 6. Lesion Age, 7. Lesion Pink	80.46%	62.09%	71.27%	70.13%
Fusion: 7 C4C risk factors 11 External Features	1. Lesion Size, 2. Lesion Colour, 3. Lesion Shape, 4. Lesion >7mm, 5. Lesion Inflamed, 6. Lesion Oozing, 7. Lesion Itch, 8. Lesion Pink, 9. Lesion Age, 10. Patient Age, 11. Patient Gender, 12. Lesion Body, 13. Moles, 14. Williams Score, 15. Sunburn, 16. Williams Group, 17. Hair Colour, 18. Freckles	**85.24%**	61.12%	**73.18%**	74.15%

Significant values are in [bold].

### Using metadata alone

We identified a new set of seven primary risk factors, termed *”C4C risk factors”*, including lesion pinkness, lesion size, lesion colour, lesion inflammation, lesion shape, lesion age, and natural hair colour. These factors are pertinent not only to melanoma but to all three main skin cancer types. In our previous metadata-based study^24^, we assessed the effectiveness of the C4C risk factors in detecting suspicious skin lesions through ensembling of ML models, comparing them to the 7PCL^7^ and Williams risk factors^25^. We achieved a sensitivity of $80.46 \pm 2.50\%$ and a specificity of $62.09 \pm 1.90\%$ in detecting suspicious skin lesions as shown in Table 2. Furthermore, fusing the *C4C risk factors* along with 11 external risk factors achieved the best performance, with a sensitivity of $85.24 \pm 2.20\%$ and a specificity of $61.12 \pm 0.90\%$.

**Fig. 11:**
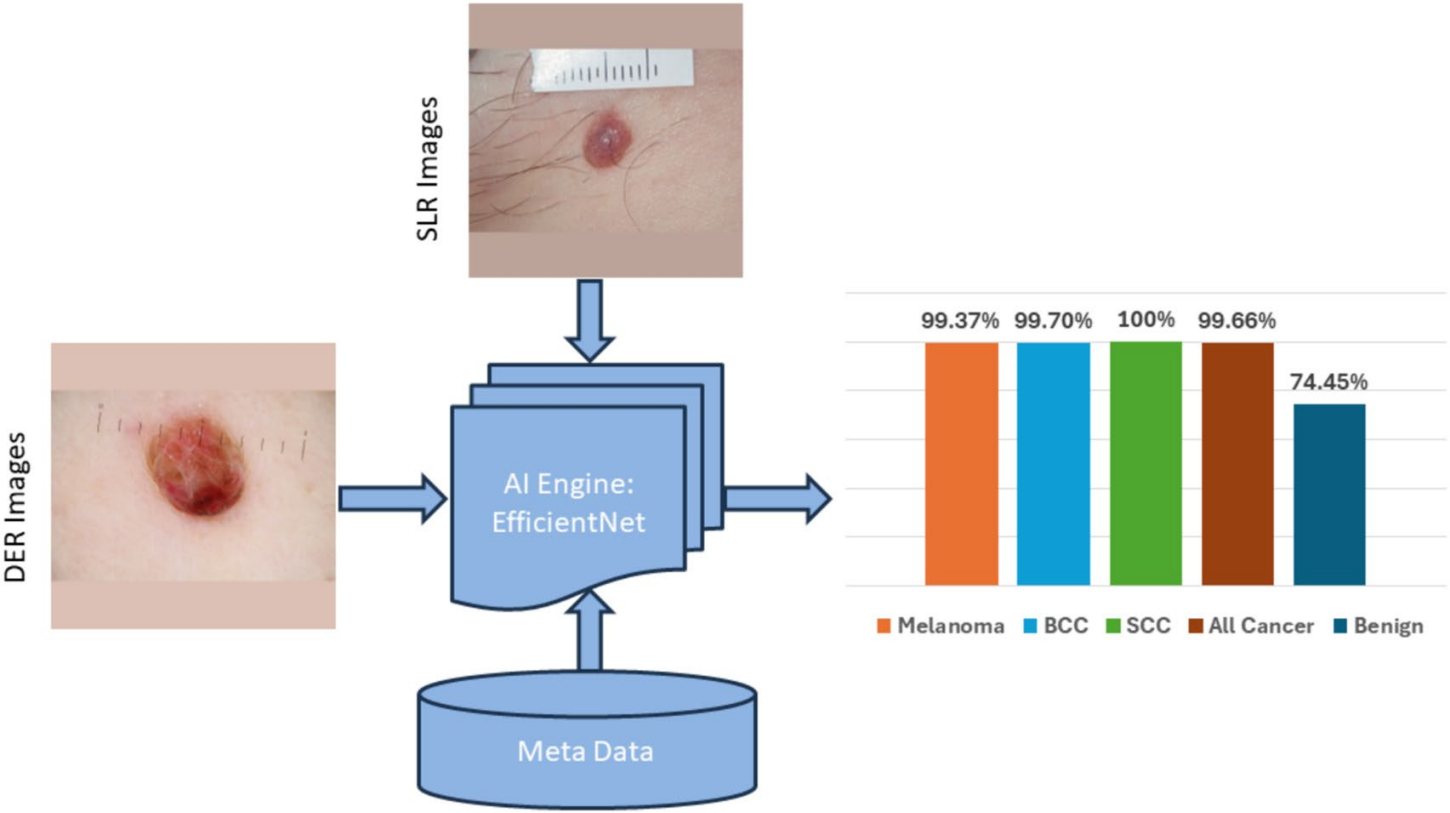
Performance of the AI-based model by fusing patient metadata and images.

### Using image data alone

The results of the AI model using image data alone are shown in Fig. 10, with AI model performance for overall skin cancer detection of 99.72 ± 1.35%, with individual results for melanoma (99.36 ± 0.72%), squamous cell carcinoma - SCC (100 ± 0%), basal cell carcinoma - BCC (99.80 ± 0.30%) and 63.22 ± 3.11% for benign (non-malignant cases).

### Image and metadata fusion

The results of the AI model using a combination of image data and metadata are shown in Fig. 11, with AI model performance for overall skin cancer detection of 99.66 ± 0.28%, with individual results for melanoma (99.36 ± 0.72%), SCC (100 ± 0%), BCC (99.50 ± 0.34%) and 74.45 ± 0.80% for benign (non-malignant cases). The incremental gain in model accuracy of 11. 23% (74.45 ± 0.80% – 63.22 ± 3.11%) for the correct classification of benign lesions is significant and will substantially reduce the number of false positives and unnecessary clinical appointments for a possible biopsy. This gain in model performance is novel and has the potential to be used during teledermatology triage.

Furthermore, to enhance the robustness of our AI model, we performed 5-fold cross-validation by varying the training data to ensure that the model performs well on unseen test data. The detailed results of the 5-fold cross-validation are consistent with those obtained from the 80/20 train/test split, as summarised in Supplementary table 1 of the Supplementary Document Section 5.

**Table 3:** Performance comparison of individual models developed and evaluated based on different data combinations.

Model	Tested on	Sensitivity	Specificity	ACC	AUC
EfficientNet–B2–DER	DER images	99.50%	63.06%	81.28%	89.43%
EfficientNet–B2–SLR	SLR images	95.48%	63.51%	79.50%	87.91%
EfficientNet–B2–DER–Meta	DER images+metadata	99.50%	74.73%	87.15%	92.20%
EfficientNet–B2–SLR–Meta	SLR images+metadata	99.33%	72.25%	85.79%	91.41%
EfficientNet–B2–DER–SLR	DER images	99.83%	69.95%	84.89%	90.53%
EfficientNet–B2–DER–SLR	SLR images	99.67%	57.25%	78.46%	87.06%
EfficientNet–B2–DER–SLR–Meta	DER images+metadata	**99.83%**	**77.71%**	**88.77%**	**92.98%**
EfficientNet–B2–DER–SLR–Meta	SLR images+metadata	99.50%	71.20%	85.35%	91.19%

Significant values are in [bold].

**Table 4:** Performance comparison of AI model outcome decision fusion along with the best individual model.

Model	Tested on	Sensitivity	Specificity	ACC	AUC
EfficientNet–B2–DER–SLR–Meta	DER images+metadata	99.83%	77.71%	88.77%	92.98%
Fusion: EfficientNet–B2–DER–SLR EfficientNet–B2–DER–SLR–Meta	SLR images DER images+metadata	**99.50%**	**82.72%**	**91.11%**	**94.06%**
Fusion: EfficientNet–B2–DER–SLR–Meta EfficientNet–B2–DER–SLR EfficientNet–B2–DER–SLR–Meta	SLR images DER images DER images+metadata	100.00%	75.38%	87.69%	92.40%
Fusion: EfficientNet–B2–DER–Meta EfficientNet–B2–DER–SLR–Meta EfficientNet–B2–DER–SLR EfficientNet–B2–DER–SLR–Meta	DER images+metadata SLR images+metadata DER images DER images+metadata	99.66%	80.12%	89.89%	93.55%
Fusion: EfficientNet–B2–SLR–Meta EfficientNet–B2–DER–Meta EfficientNet–B2–DER–SLR–Meta EfficientNet–B2–DER–SLR EfficientNet–B2–DER–SLR–Meta	SLR images+metadata DER images+metadata SLR images+metadata DER images DER images+metadata	99.83%	75.88%	87.86%	92.55%

Significant values are in [bold].

### AI decision fusion

Table 3 summarises the performance of individual models developed and evaluated using various data combinations. The EfficientNet–B2–DER model, when tested on DER images, achieved a sensitivity of 99.50 ± 1.18%, specificity of 63.06 ± 2.80%, and 81.28 ± 2.44% balanced accuracy. Sensitivity decreased to 95.48 ± 1.85% for the EfficientNet–B2–SLR model when tested on SLR images. Adding metadata to the EfficientNet–B2–DER–Meta model significantly enhanced performance, achieving 99.50 ± 1.18% sensitivity, 74.73 ± 0.92% specificity, and 87.15 ± 1.80% balanced accuracy. The best results were obtained with the EfficientNet–B2–DER–SLR–Meta model tested on DER images with metadata, yielding 99.83 ± 0.49% sensitivity, 77.71 ± 0.66% specificity, and 88.77 ± 1.63% balanced accuracy. Overall, models incorporating metadata with both DER and SLR images performed better on DER images than on SLR images.

Table 4 presents a comparison of AI model performance using outcome decision fusion alongside the best individual model results. A majority voting strategy was applied to determine if a test sample fell into suspicious or non-suspicious categories. The fusion of decisions from the EfficientNet–B2–DER–SLR and EfficientNet–B2–DER–SLR–Meta models, when tested on SLR images and DER images with metadata, respectively, resulted in a notable increase in specificity, reaching 82.72 ± 1.64% (up from 77.71 ± 0.66%), compared to the best-performing single model, EfficientNet–B2–DER–SLR–Meta. The second-best fusion involved combining decisions from EfficientNet–B2–DER–Meta, EfficientNet–B2–DER–SLR–Meta, EfficientNet–B2–DER–SLR, and EfficientNet–B2–DER–SLR–Meta models, yielding 99.66 ± 0.28% sensitivity, 80.12 ± 2.10% specificity, and a balanced accuracy of 89.89 ± 2.28%. Although the fusion of EfficientNet–B2–DER–SLR–Meta, EfficientNet–B2–DER–SLR, and EfficientNet–B2–DER–SLR–Meta achieved perfect sensitivity (100 ± 0%), specificity fell to 75.38 ± 2.15%. Thus, the fusion of EfficientNet–B2–DER–SLR and EfficientNet–B2–DER–SLR–Meta offered the best balance while adding more models increased computational complexity without enhancing performance.

### Benchmarking

To compare the sensitivity of our AI model with that of HCP assessments, we used benchmark data from two Cochrane Database Systematic Reviews^35,36^. These reviews provide a comparison table for visual inspection with or without dermoscopy to detect any skin lesion requiring excision, aligning closely with our skin cancer diagnosis pathway, which covers patients presenting with symptoms of the three major skin cancer types: melanoma, BCC, and SCC. The Cochrane Reviews calculate sensitivity values at a fixed specificity of 80% to allow consistent interpretation across studies, reporting a 96% sensitivity for in-person skin cancer detection using dermoscopy. The study^37^ proposed a CNN-based metadata processing block (Metablock) comprising 21 meta-features: age, sex, anatomical region, cancer history, skin phototype, family background, along with 2298 clinical images captured using a smartphone and achieved 70% balanced accuracy that is higher than the feature concatenation-based method MetaNet (balanced accuracy, 66.20%). By comparison, our AI model achieves a sensitivity of 98.75% for all skin cancers at a specificity of 80.37%, outperforming the published in-person evaluations, as shown in Table 5. Additionally, we benchmarked our model against Skin Analytics (SA), a UK-based company collaborating with the NHS on skin cancer detection^38^. Unlike the SA model, which analyses only dermoscopic images, our multi-modal AI model demonstrated superior accuracy in detecting melanoma, BCC, and SCC and in correctly classifying benign lesions, as presented in Table 5.

**Table 5:** Performance evaluation of the proposed AI model by benchmarking with the skin analytics model and cochrane database systematic reviews.

Lesion type	HCP^35,36^ (80% fixed specificity)	Skin analytics^38^	C4C (ours)	C4C (ours) (80% fixed Specificity)
Melanoma	–	95%	99.37%	98.10%
BCC	–	98%	99.50%	98.79%
SCC	–	97%	100%	100%
All skin cancer	96%	97%	**99.66%**	**98.75%**
Benign	80%	73%	**74.45%**	**80.37%**
AUC	–	–	85.61%	89.12%

Significant values are in [bold].

**Fig. 12:**
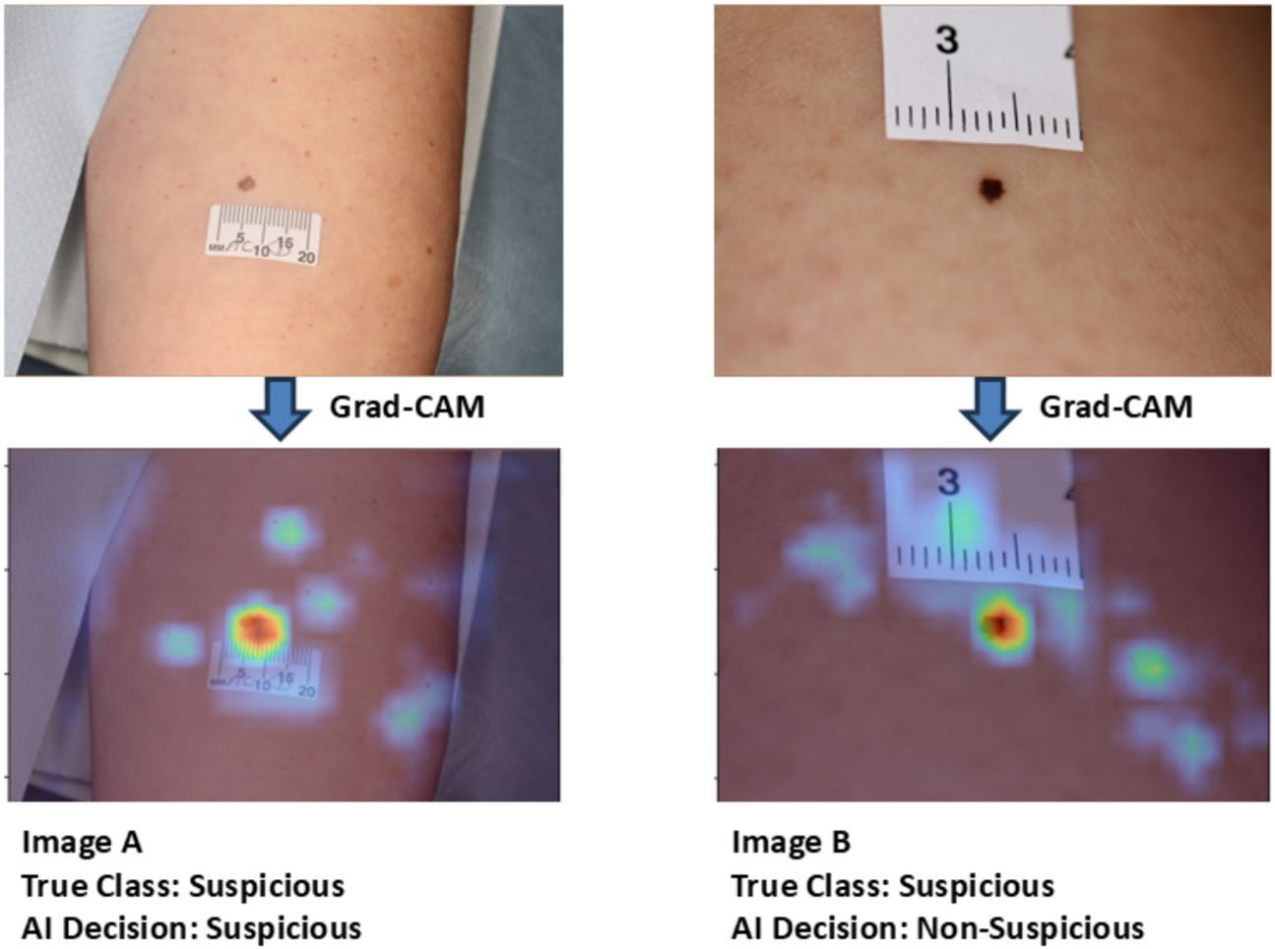
Heatmap generation from the last layer of EfficientNet–B2 using Grad-CAM to check whether the model is focused on the correct lesion area.

**Fig. 13:**
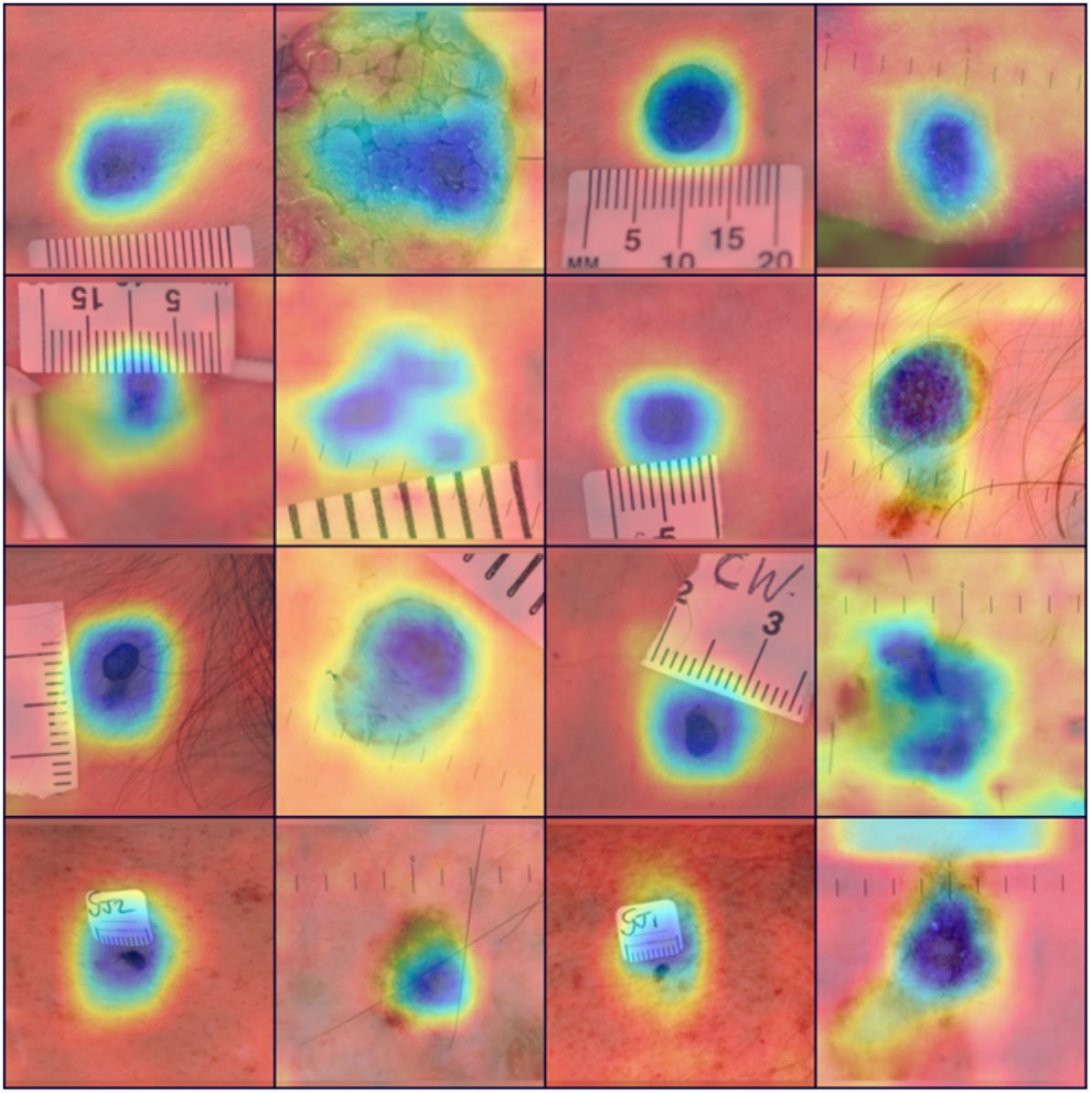
Adoption of the scaled dot product attention mentioned in the study^39^ to force the AI model to focus on the correct lesion area and ignore artefacts such as rulers.

### Explainability of AI decision

X-AI has the potential to explain a transparent decision-making process to the HCP^40^. An attempt was made to explain the decision-making of the AI model by adapting different attention mechanisms. We wanted to investigate whether the model was focusing on the correct lesion area or not during decision-making. We used Grad-CAM^41^ to produce the heat map of the last layer of the AI model and, although the model does focus on the suspicious lesion, it may also focus on other areas or artefacts such as rulers, as shown in Fig. 12. Our AI model correctly classified Image A, Image B was misclassified. Upon heatmap investigation, we found that the AI model was giving significant attention to the artefacts (ruler) along the lesion area in Image B, which led to misclassification. Therefore, the adoption of soft-attention modules to guide the AI model in focusing on the correct lesion area is crucial. To tackle this issue, we adapted soft attention mechanisms inspired by the DINO model, a vision transformer model proposed by the Facebook AI research team^39^, in order to force the AI model to focus on the correct lesion area by adoption of this attention mechanism. This investigation is ongoing, and a snapshot of promising preliminary results of the scaled dot product attention is shown in Fig. 13 for illustrative purposes.

There are some limitations with this study. We utilised lesion ratings (Suspicious or Non-suspicious) classified by our in-house expert as the ground truth instead of biopsy results. Although biopsy results were available, we have a limited number of lesions that went for biopsy (only 10% of lesions undergo biopsy). If we only use biopsy results as the target variable, the data size will reduce significantly (90%). As a result, the lesion rating was used here as the target variable rather than the biopsy results. The ultimate goal is to use AI as a clinical decision aid for the classification of suspicious or non-suspicious skin lesions during teledermatology triage. We implemented hair removal and lesion detection as a pre-processing step. We observed that the reconstructed image quality differs from the original raw image in terms of image size, resolution, and visual appearance. In our future research, we aim to adapt a quality check monitor to ensure reconstructed images do not lose vital information from lesion characteristics.

In an attempt to further improve AI model performance, we adopted six conventional feature extraction techniques- Hu moments, Zernike moments, Haralick features, binary and colour histograms, and ABCD features. The goal of feature extraction was to integrate the extracted features with the AI’s interpretation of the image. When we fused the extracted conventional features along with image data, performance did not improve as compared to the fusion of metadata and images. In the future, we plan to extensively investigate feature extraction and selection followed by fusion to attempt further model performance improvement.

## Conclusion

The fusion of multi-modal data comprising patient metadata and skin lesion images, followed by applying advanced AI techniques, has great potential to detect suspicious skin lesions at an early stage during teledermatology triage. With a reduction in patient referrals for possible biopsy, waiting times for skin cancer diagnosis and treatment will be shortened, resulting in improved outcomes. In this study, we devised an AI framework based on multi-modal data input for skin lesion classification, which outperformed the existing state-of-the-art results reported by Skin Analytics, as well as published in-person evaluation. This study contributed to high-quality multi-modal data collection followed by AI model development and AI decision fusion for suspicious skin cancer detection. Fusing patient metadata along with image data significantly improved the AI model performance as compared to using image data alone. We further attempted to explain AI decisions by adding a post-processing module that uses Grad-CAM to generate heatmaps, which show where an AI model focuses on during decision-making to decide whether a skin lesion belongs to suspicious or non-suspicious categories. In our future research, we are extending our investigation by adopting a soft-attention that forces an AI model to focus only on the lesion area and ignore artifacts such as rulers present in the images, which we believe will further boost the performance of skin cancer detection cost-effectively.

## Supplementary Information

Supplementary Information.

## Data Availability

The dataset used for developing and evaluating the AI model in this study is not publicly available due to data governance policies and its inclusion in a pending patent application (UK IPO Ref: 2415479.1). However, anonymised data may be made available to academic researchers strictly for non-commercial replication purposes under a formal data-sharing agreement with Check4Cancer. Requests for data access may be directed to Professor Gordon Wishart (Check4Cancer Ltd).

## Abbreviations

NHS: National Health Service
AI: Artificial intelligence
NMSC: Non-melanoma skin cancer
BCC: Basal cell carcinoma
SCC: Squamous cell carcinoma
7PCL: 7-point checklist
ML: Machine learning
KNN: K-nearest neighbour
SVM: Support vector machine
DL: Deep learning
CNN: Convolutional neural networks
ISIC: International Skin Imaging Collaboration
ViTs: Vision transformers
C4C: Check4Cancer
DSLR: Digital single-lens reflex camera
DER: Dermoscopic camera
Grad-CAM: Gradient class activation map
ACC: Balanced accuracy
AUC: Area under the curve
HCP: Health Care Professional
X-AI: Explainable AI
DINO: Self-distillation with no labels
