# Supplementary material for: Advancing skin cancer detection through deep learning and fusion of patient metadata and skin lesion images
Source: Sci Rep. 2026 Jan 13;16:1968. doi: 10.1038/s41598-025-26392-4 (PMC12808132; doi:10.1038/s41598-025-26392-4)
Supplement: Supplementary file 1 — Supplementary Information. [file 41598_2025_26392_MOESM1_ESM.pdf]

5 Supplementary Document

Supplementary table 1. The multi-fold cross-validation performances of the developed AI model.

Fold	#Training Images	#Testing Images	Sensitivity	Specificity	ACC	AUC
1	Total: 23500 images<br>Non-suspicious: 20000<br>Suspicious: 3500	Total: 9182 images<br>Non-suspicious: 7988<br>Suspicious: 1194	99.58%	72.51%	86.05%	86%
2	Total: 23500 images<br>Non-suspicious: 20000<br>Suspicious: 3500	Total: 9182 images<br>Non-suspicious: 7988<br>Suspicious: 1194	99.32%	72.26%	85.80%	85.62%
3	Total: 23060 images<br>Non-suspicious: 20000<br>Suspicious: 3064	Total: 9182 images<br>Non-suspicious: 7988<br>Suspicious: 1194	99.49%	72%	85.66%	85.67%
1+2	Total: 47000 images<br>Non-suspicious: 40000<br>Suspicious: 7000	Total: 9182 images<br>Non-suspicious: 7988<br>Suspicious: 1194	99.58%	71.51%	85.54%	85.57%
1+2+3	Total: 70064 images<br>Non-suspicious: 60000<br>Suspicious: 10064	Total: 9182 images<br>Non-suspicious: 7988<br>Suspicious: 1194	99.66%	74.45%	87.06%	87.10%
Overall Average			99.53%	72.55%	86.02%	85.99%
